# ﻿*Hedyotiskonhanungensis* (Rubiaceae): A new species from the central highlands of Vietnam

**DOI:** 10.3897/phytokeys.221.95895

**Published:** 2023-03-09

**Authors:** Bui Hong Quang, Khang Sinh Nguyen, Tuan Anh Le, Le Thi Mai Linh, Quoc Luan Nguyen, Duy Hoang Vu Ngo, Lei Wu, Suman Neupane

**Affiliations:** 1 Institute of Ecology and Biological Resources, Vietnam Academy of Science and Technology, 18 Hoang Quoc Viet, Cau Giay, Hanoi-10000, Vietnam Institute of Ecology and Biological Resources, Vietnam Academy of Science and Technology Hanoi Vietnam; 2 Graduate University of Science and Technology, Vietnam Academy of Science and Technology, 18 Hoang Quoc Viet, Cau Giay, Hanoi-10000, Vietnam Graduate University of Science and Technology Hanoi Vietnam; 3 Mien Trung Institute for Scientific Research, Vietnam National Museum of Nature, VAST, 321 Huynh Thuc Khang, Thua Thien Hue-49000, Vietnam Mien Trung Institute for Scientific Research, Vietnam National Museum of Nature Thua Thien Hue Vietnam; 4 Kon Ka Kinh National Park, Gia Lai-61000, Vietnam Kon Ka Kinh National Park Gia Lai Vietnam; 5 College of Forestry, Central South University of Forestry and Technology, Changsha 410004, China Central South University of Forestry and Technology Changsha China; 6 Department of Biological Sciences, 1112G Biology Building, Murray State University Murray, KY 42071, Murray, USA Murray State University Murray United States of America

**Keywords:** Gia Lai Province, *Hedyotis-Oldenlandia* complex, Indochina, phylogenetics, Spermacoceae, taxonomy

## Abstract

A new species of *Hedyotis* L. (Rubiaceae), *Hedyotiskonhanungensis* B.H. Quang, T.A. Le, K.S. Nguyen & Neupane, is described and illustrated from the central highlands of Vietnam based on morphological and phylogenetic evidence. The new species belongs to the morphologically diverse tribe Spermacoceae (ca. 1000 species) of the family Rubiaceae, which is represented by 70–80 species in Vietnam. The phylogenetic analysis, based on four DNA regions (ITS, ETS, petD, rps 16), confirms the new species’ placement within the genus *Hedyotis* – one of the largest genera in the tribe, comprising ca. 180 species across Asia and the Pacific. *Hedyotiskonhanungensis* is morphologically distinct from all southeastern Asian *Hedyotis* L. in its set of traits such as leaf type (shape and thickness), growth habit, and floral parts (color of inflorescence axis and the shape of calyx lobes). The new species shows similarities with *Hedyotisshenzhenensis*, *H.shiuyingiae*, and *H.yangchunensis* from China in its herbaceous habit, fleshy ovate leaf blades, and dark purple floral parts, but it is phylogenetically distinct and can be distinguished from them by the following combination of morphological traits: habit with slightly smaller stature (<25 cm), broadly ovate or deltoid stipules with cuspidate apex and entire margin, and ovate or nearly ovate calyx lobes.

## ﻿Introduction

The Asian-Pacific genus *Hedyotis* L. (ca. 180 species) lies within a polymorphic, mainly herbaceous tribe Spermacoceae (ca. 1000 species) of the family Rubiaceae. In Vietnam, this tribe is represented by 70–80 species belonging to the genera *Dimetia* (Wight & Arn.) Meisn., *Debia* Neupane & N.Wikstr., *Exallage* Bremek., *Leptopetalum* Hook. & Arn., *Hedyotis* L., *Involucrella* (Benth. & Hook.f.) Neupane & N.Wikstr., *Neanotis* W.H.Lewis, *Oldenlandia* L., and *Spermacoce* L. (Govaerts et al. 2022). The genus *Hedyotis* and its related genera that form the *Hedyotis-Oldenlandia* complex have a long history of taxonomic confusion and disagreement due to the use of inconsistent and overlapping characters in the generic delimitations. The genus *Hedyotis* is currently treated in a narrow sense based on the molecular phylogenies (Guo et al. 2013; Wikström et al. 2013; Neupane et al. 2015) where members of it are united by their predominantly shrubby to tree-like habit, septicidal capsules, flattened seeds, and tropical upland distributions in Asia and the Pacific. The species of this genus are mostly found in the mid or high-elevation slopes (to 4000 m a.s.l.) of Asia (Sri Lanka, southern India, SE China, Indochina, Malesia), Papuasia, and NW Pacific (Micronesian islands). In Vietnam there are about 20 species reported in the flora of this country (Pitard 1922; Pham 2003; Tran 2005; Do et al. 2013; Govaerts et al. 2022) but the actual number could be as high as 40 species since many species listed under *Oldenlandia* share the diplophragmous capsule characteristic of *Hedyotis* (pers. obs.).

During a botanical field survey in Kon Ha Nung Biosphere Reserve (UNESCO 2021) of the highlands of Central Vietnam, we came across a population of Spermacoceae (collection number *LTA 531*) with the following morphological features: perennial herbs, opposite obovate to nearly oval leaf blades, interpetiolar entire stipules with cuspidate apex, 4-merous flowers, bilobed stigmas, inferior ovary, and many-seeded, diplophragmous capsules with persistent ovate or nearly oval calyx lobes. The morphological features, specifically the presence of diplophragmous capsules, observed in the new population of Spermacoceae align with the diagnostic characteristics of the genus *Hedyotis* as outlined in Wikström et al. (2013) and Neupane et al. (2015). Following a thorough review of the taxonomic literature on the genus *Hedyotis* in Vietnam and surrounding regions, including Pitard (1922), Fukuoka (1970), Pham (2003), Tran (2005), Chen and Taylor (2011), Wikström et al. (2013), and Neupane et al. (2015), as well as a comprehensive morphological comparison with closely related species such as *Hedyotisshenzhenensis* T.Chen, *H.shiuyingiae* T.Chen, and *H.yangchunensis* Ko & Zhang, and utilizing phylogenetic analysis to determine its placement within the Spermacoceae, we have determined that our specimens represent a previously undescribed species within the genus *Hedyotis*. This species is hereby named *Hedyotiskonhanungensis*.

## ﻿Materials and methods

### ﻿Morphological study

Our collected specimens were compared with all described species from southeast Asia and southern China by studying relevant literature and examining digital herbarium images. Morphological characters were recorded using Nikon SMZ745/SMZ745T stereoscopic microscope and photographs of vegetative and floral parts were taken both in the field and from the samples preserved in 70% ethanol using Canon EOS 7D. The type specimens have been stored in the following three herbaria (acronyms follow Thiers 2022, continuously updated): Vietnam National Museum of Nature (**VNMN**), Institute of Ecology and Biological Resources, Vietnam Academy of Sciences and Technology (**HN**), and Kon Ka Kinh National Park.

### ﻿Phylogenetic analysis

To establish its phylogenetic position within the tribe Spermacoceae, total genomic DNA was extracted from silica-dried material with the DNeasy Plant Kit (Qiagen, Valencia, California, U.S.A.). Four DNA regions (nuclear genome: ITS, ETS; plastid genome: *petD*, *rps16*) that were used in our earlier studies (Neupane et al. 2015, Neupane et al. 2017), were selected for amplification from this sample. Amplifications were performed in a 25 μl reaction mixture composed of 1 μl of each primer (10 μM), 1 μl of DNA template, 12.5 μl of GoTaq Green Master Mix (Promega, Madison, Wisconsin, U.S.A.), 9.5 μl of water. The amplification protocol for nuclear and chloroplast regions follows Kårehed et al. (2008) and Groeninckx et al. (2009) respectively. Amplified PCR products were purified using ExoSAP-IT PCR Product Cleanup (Thermo Fisher Scientific) following the manufacturer’s protocols and sequenced at Apical Scientific Company for sequencing (Selangor, Malaysia).

We added the DNA sequences of our sample to our existing DNA data matrix (Suppl. material 1). The GenBank accession numbers for our sample were OQ401065, OQ401066, OQ458733, and OQ401067, which correspond to the rps16, petD, ITS, and ETS regions, respectively. We also included additional sequences representing the ITS, petD, and rps16 regions for *Hedyotisyangchunensis*, *Hedyotisshenzhenensis*, and *Hedyotisshiuyingiae*, obtained from Guo et al. (2013) and available in GenBank. Each of these DNA regions was aligned using MAFFT v.7 (Katoh and Standley 2013) and concatenated into a single matrix Suppl. material 2) to infer the phylogeny. Phylogenetic analysis was conducted on the combined matrix under the maximum likelihood (ML) framework with four partition schemes (ITS, ETS, petD, rps16) for their own substitution model (GTR+GAMMA) parameters using RAxML v.8.2.12 (Stamatakis 2014). The RAxML tree search and bootstrap analysis (1000 rapid bootstrap replicates for clade support) were conducted on CIPRES (Miller et al. 2010).

## ﻿Results

### ﻿Molecular phylogeny

**Figure 1.**
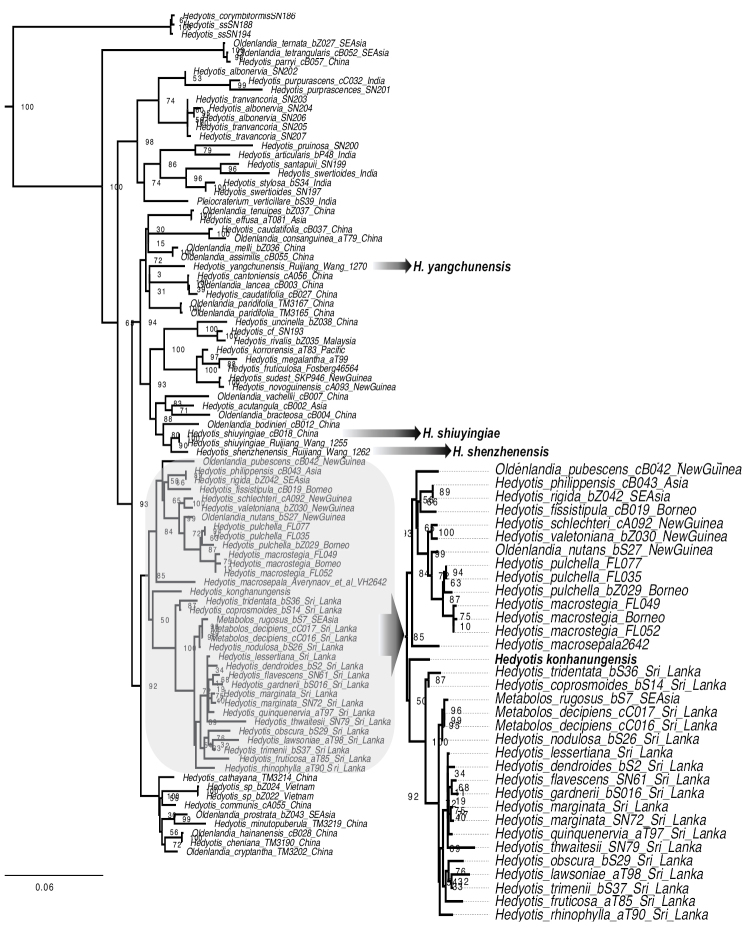
Maximum likelihood phylogenetic tree of *Hedyotis* based on the combined nuclear (ITS, ETS) and plastid (*petD*, *rps16*) data. The taxa discussed in the paper and the new species are in bold. Values at the nodes represent bootstrap support (BS).

The Maximum likelihood tree obtained from RAxML analysis on the concatenated data confirms the position of our sample from Vietnam within *Hedyotis* and distinct from *H.yangchunensis*, *H.shenzhenensis*, and *H.shiuyingiae* (Fig. 1, Suppl. material 3) The new species is nested within the clade (BS = 85%) representing *Hedyotis* from Sri-Lanka, southeast Asia (primarily Borneo), and New Guinea. The new species appears sister to the Sri Lankan clade, however, this phylogenetic position is considered unresolved due to weak bootstrap support (BS = 50%).

### ﻿Taxonomic treatment

#### ﻿ Hedyotis konhanungensis

Taxon classificationPlantaeGentianalesRubiaceae

B.H.Quang, T.A.Le, K.S.Nguyen & Neupane sp. nov.

AEE11259-0F75-5C0A-ACC1-758BD4048DB5

urn:lsid:ipni.org:names:77315486-1

**Figure 2.**
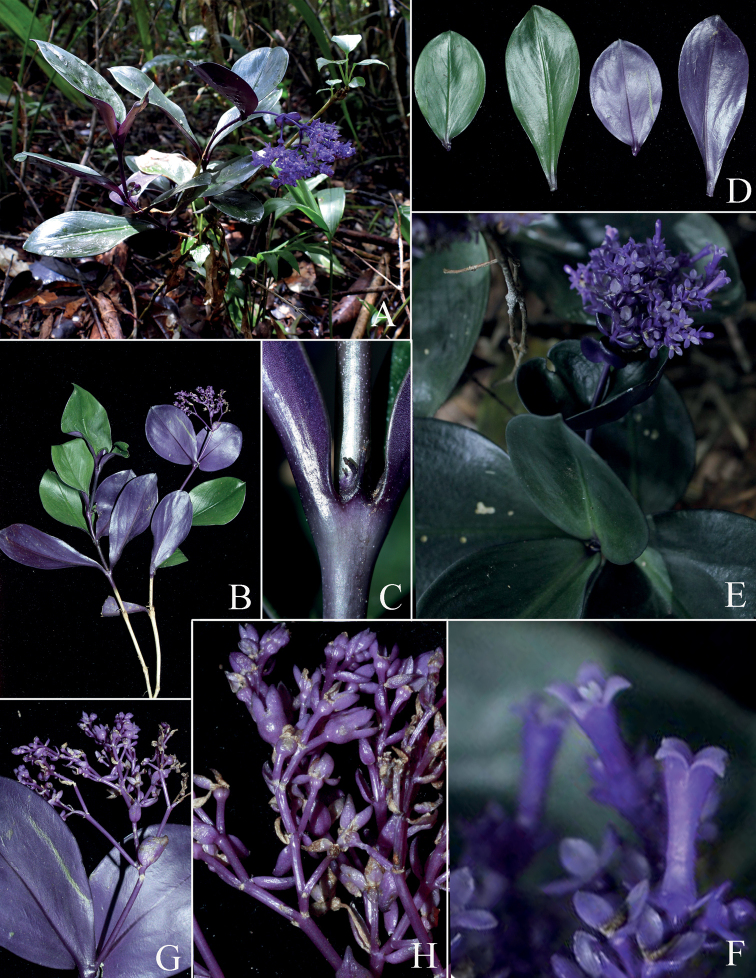
*Hedyotiskonhanungensis***A** habitat **B** habit **C** stipule **D** adaxial and abaxial leaf surfaces **E** inflorescences top view **F** long-styled and short-styled flower **G** infructescence **H** fruiting branch. Photos by Q.L. Nguyen and B.H. Photos by Quang, from *Luan et al. LTA 531*.

**Figure 3.**
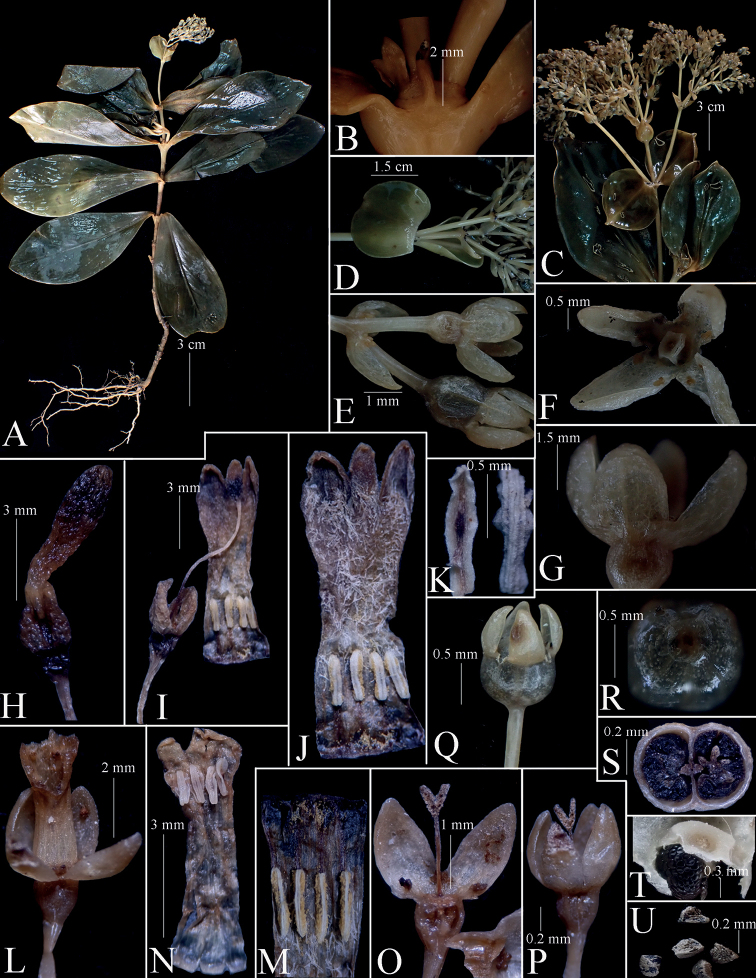
*Hedyotiskonhanungensis***A** habit **B** stipule **C** inflorescence **D** floral bracts **E** bracteoles and fruits **F, G** calyx top and side views **H–K** long-styled flower, entire and open showing pistil, corolla and anthers **L–P** short-styled flower, dissection showing calyx, corolla, anthers and pistil **Q–T** fruit, entire and dissection **U** seeds. Photos and design by B.H. Quang from *Luan et al. LTA 531*.

Figs 2 , 3 , Table 1

##### Type.

Vietnam. Central Highlands of Vietnam, Kon Ha Nung Biosphere Reserve, Gia Lai province: K’Bang District, Kon Pne Commune, 14°20'53"N, 108°20'48"E, primary evergreen forest slopes at elevation 1150 m a.s.l., 28 November 2021, Nguyen Quoc Luan, Ngo Duy Hoang Vu & Le Tuan Anh LTA 531 (holotype: VNMN!; isotypes: HN!).

##### Diagnosis.

*Hedyotiskonhanungensis* is similar to *H.shenzhenensis*, *H.shiuyingiae* and *H.yangchunensis* from southeastern China (Guangdong and Hongkong) in the morphology of the leaf blades, floral bracts, dichasial cymes, and fruits, but differs from them by its broadly ovate or deltoid (vs. triangular or broadly triangular) stipules with entire (vs. hairy or lacerated) margins and cuspidate (vs. acute) apex, suborbicular or broadly oval (vs. subovate or ovate to lanceolate) lowest floral bracts, ovate or nearly oval (vs. triangular or subulate to lanceolate) persistent calyx lobes on fruits, and stamens in long-styled flowers inserted in lower ¼ or near the base (vs. at the middle or near the mouth) of the corolla tube (Table 1).

**Table 1.** Morphological comparison of *Hedyotiskonhanungensis* with its putative closest allies. The characters of *H.shenzhenensis* T.Chen, *H.shiuyingiae* T.Chen and *H.yangchunensis* W.C.Ko & Zhang are taken from Chen (2007, 2008), and Gao (1995) and their type specimens respectively.

Morphological characters	*H.konhanungensis*	*H.shenzhenensis*	*H.shiuyingiae*	*H.yangchunensis*
Plant height, including the inflorescences (cm)	15–25	20–40	15–35	30–39
Stem	terete or subterete, 8–18 cm long	terete or subterete,1–2 cm long	slightly tetragonal, 10–22 cm long	tetragonal or subterete, 15–30 cm long
Leaf arrangement	evenly spaced along the stem	fascicled on short rosette-like stem	evenly spaced along the stem	evenly spaced along the stem
petiole length (mm)	4–6	0.5	5–10	15–20
blade shape and size (cm)	obovate to oval, 6–10 × 2–4	elliptic, oblong-elliptic or obovate, 8.5–15 × 5–9	broadl, lanceolate or obovate-lanceolate, 2–19 × 1.4–8	narrowly elliptic, oblanceolate or elliptic-oblong, 3–12 × 1–4.5
Stipule shape and size (mm)	broadly ovate or deltoid, apex cuspidate, margin entire, 2.5–3 × 5–6	triangular, apex acute, margin shortly hairy, 3–5 × 5–10	triangular, apex acute, margin dense trichomes, 4–8 × 5–10	triangular, apex and margin lacerate or deeply divided into several linear lobes, ca. 16 × 8
Inflorescence	Longistylous flowers lax or not congested into a head-like inflorescence	Longistylous flowers lax or not congested into a head-like inflorescence	Longistylous flowers congested into a head-like inflorescence	Longistylous flowers congested into cymose or capitate inflorescence
Peduncle length (cm)	2–4	10–18	10	3–4
Lowest floral bracts	suborbicular or broadly oval, 2–2.5 × 1.5–2 cm	subovate, 2.5–3 × 1.8–2 cm or sometimes larger	ovate to lanceolate, 0.6–2.5 × 0.2–1 cm	ovate, ca. 1.5 × 0.5–0.6 cm
Calyx lobes	ovate or nearly oval, 1.7–2.2 × 0.8–1.4 mm	subulate, ca. 1.5 mm long	subulate, ca. 3 mm long	unknown
Corolla				
outer colour	bluish purple	white	purplish white	unknown
tube dimension and shape	6–7 × 1–1.5 mm, slightly enlarged both ends	ca.3 × 1.5–1.8 mm, slightly narrower near mouth	tube ca. 4.5 × 2–2.5 mm, enlarged at mouth	unknown
Stamens of long-styled flowers	inserted in lower ¼ (or near the base) of corolla tube	inserted in upper ¼ (or near the throat) of corolla tube	inserted in ½ (or middle) of corolla tube	unknown
Stamens of short-styled flowers	inserted in the upper ¼ (or near the throat) of corolla tube;	inserted near the throat of corolla tube;	inserted near base of corolla tube;	unknown
	filaments ca. 0.2 mm long, not exserted beyond corolla mouth	filaments ca. 0.8 mm long, exserted beyond corolla mouth	filaments ca. 0.3 mm long, not exserted beyond corolla mouth	
Persistent calyx lobes on fruits	ovate or nearly oval, 1.8–2.3 × 0.8–1.5 mm, unveined	narrowly triangular or subulate, 1.2–2 × 0.5–0.7 mm, unveined	narrowly triangular or subulate, 3–4 × ca.1 mm, unveined	lanceolate, 3–6 × 1.2–3 mm, veined

##### Description.

Perennial herbs, erect, 15–25 cm tall, completely glabrous. ***Stem*** simple, rarely branched, terete, 8–18 cm long, 5–7 mm in diam.; internodes 1–1.5 cm long. ***Leaves*** 5–7 pairs per stem, decussate, evenly spaced along the stem, abaxial side dark green, adaxial side glossy, dark purple to purplish black; petioles 4–6 mm long; blade flattened, thick, fleshy (subcoriaceous when dried), obovate-lanceolate to nearly oval, 6–10 × 2–4 cm; base decurrent or cuneate; apex broadly acute or obtuse; midrib depressed adaxially and prominent abaxially; 4–5 secondary veins on each side of the midrib, inconspicuous on adaxial side. ***Stipule*** interpetiolar, fused to leaf bases or very shortly around stem, broadly ovate or deltoid, 2.5–3 mm long, 5–6 mm wide at base, flattened, dark purple outside; apex cuspidate or aristate, with aristae 3–4 mm long and 0.7–0.9 mm wide; margins entire. ***Inflorescence*** terminal, a compound dichasial cyme, 5–7 cm long, with 3–4 orders of branching, purplish, 30–50-flowered. Peduncle terete, 2–4 cm long; bracts subtending the basal branches of the inflorescence leaf-like, suborbicular or broadly oval, slightly concave, 2–2.5 × 1.5–2 cm, with apex rounded to broadly acute or obtuse, abaxially dark purple to dark bluish purple, pale green adaxially; bracts subtending the upper inflorescence branches smaller, ovate or lanceolate, 0.8–1.5 × 0.5–1 cm, with acute apex. Pedicels terete, 3–6 mm long, usually bluish purple or purplish, bracteolate or ebracteolate; bracteoles narrowly ovate or lanceolate, somewhat concave, ca. 2.5 mm × 1.5 mm, with apex broadly acute or obtuse. ***Flowers*** 4-merous, distylous. ***Calyx*** hypanthium, bluish purple, cupular, 0.8–1.2 mm long and wide, glossy and glabrous; lobes 4, sub-equal, ovate or nearly oval, somewhat longitudinally concave, 1.7–2.2 mm long, 0.8–1.4 mm wide, broadly acute to obtuse at apex, adaxially white or purplish white, abaxially bluish purple. ***Corolla*** narrowly infundibuliform or tubular, bluish purple outside, white pubescent inside; tube 6–7 mm long, 1–1.5 mm wide, slightly enlarged at both ends; lobes 4, strongly reflexed, triangular-ovate, 1.5–1.8 × 1.2–1.5 mm, acute, adaxially white or purplish white, sparsely puberulent near base on adaxial surface. ***Longistylous flowers***: stamens inserted in the lower ¼ of corolla tube, 1.5–2 mm above the base of tube; filaments very short, ca. 0.2 mm long; anthers linear, 1–1.2 × 0.2 mm, dorsifixed, introrse; style filiform, 5.5–6.5 mm long, white, glabrous, 2-parted; lobes oblong, 0.6–0.7 mm long, abaxially minutely papillose, pure white, slightly exserted beyond the corolla mouth; ovary inferior, 2-locular, with numerous ovules, placentation axile. ***Brevistylous flowers***: stamens inserted on the upper ¼ of corolla tube, 1–1.5 mm below the corollas mouth; filaments and anthers similar to those in the long-styled flowers; style filiform, 1.5–1.8 mm long, 2-parted; lobes oblong, 0.6–0.7 mm long, densely papillose abaxially. ***Disks*** fleshy, ring-shaped, concave in the center, glabrous, white. ***Fruits*** capsular, nearly cupuliform with slightly concave top, 2.5–3 mm in diam., glabrous, purplish, crowned by the persistent, unveined, oval or nearly oval calyx lobes. Seeds many, black, irregularly angular, reticulate, minute, 0.3–0.5 mm long.

##### Additional specimens examined

**(Paratypes).** Vietnam. Central Highlands of Vietnam. Kon Ha Nung Biosphere Reserve, Central Highlands of Vietnam, Gia Lai province: K’Bang District, Kon Pne Commune, 14°20'43.06"N, 108°20'38.33"E, 906 m a.s.l., 26 March 2022, *Bui Hong Quang et al. BHQ 453* (HN, and herbarium of Kon Ka Kinh National Park).

##### Etymology.

This species is named after the “Kon Ha Nung Biosphere Reserve” where it was discovered.

##### Vernacular name.

Vietnamese: An điền Kon Hà Nừng

##### Phenology.

Flowering in October to November, fruiting from November to December.

##### Distribution and ecology.

*Hedyotiskonhanungensis* is recorded only from the type locality in the Kon Pne Commune of the Central Highlands of Vietnam, which is part of the Annamite Range. This range is known for its rich biodiversity and high number of endemic species (Averyanov et al. 2003). The species grows in the understorey of the evergreen forests in the valleys or on flat areas to slopes of sandstone mountains. Within its occupancy areas, the new species was associated with some shrubs or herbs such as *Pavettabauchei* Bremek., *Lasianthusbiflorus* (Blume) M.Gangop. & Chakrab., *Staurogyne* sp., *Popowia* sp., *Huperzia* sp.

## ﻿Discussion

The genus *Hedyotis*, as currently circumscribed, corresponds to a primarily woody genus characterized by “diplophragmous” capsules (septicidally dehiscent capsules that separate into two distinct valves, as described by Wight and Arnott in 1834) and “fruiticosa type” seeds (dorsiventrally flattened seeds with a ventral hilar ridge topped by a punctiform apical hilum, as described by Terrell and Robinson in 2003). The members of this hyperdiverse genus (approximately 180 species) primarily occupy the mountains of Asia and the Micronesian Islands (in the northwestern Pacific). The molecular dating analysis suggests that *Hedyotis* split from its sister lineages of approximately 10 species (the African/Malagasy *Agathisanthemum* group and the African-tropical Asian-North American *Edrastima* group) nearly 27 million years ago (Neupane et al. 2017). The phylogenetic comparative analysis of evolutionary correlations shows that the evolution and diversification of the hyperdiverse *Hedyotis* along tropical mountain orogeny are strongly linked to the formation of a woody habit and many narrow endemic species (Neupane et al. 2017). The new species *Hedyotiskonhanungensis*, found in the mountains of central Vietnam, is another example of a tropical montane species in this genus. This species is resolved within the clade containing other southeast Asian species and is unique among the Indochinese *Hedyotis* in its thick and fleshy oval leaves and completely dark purple floral parts (calyx and corolla) including the inflorescence stalk. Despite the geographic distance, its morphological similarity with Chinese members and non-monophyletic group of *H.shenzhenensis* and *H.shiuyingiae* with *H.yangchunensis* (Fig. 1) suggests the independent evolution of fleshy leaves and somewhat stunted growth habit among these species. Here we provide both morphological and molecular evidence to confirm the novelty of this taxon and propose it as species new to science.

## Supplementary Material

XML Treatment for Hedyotis konhanungensis

## Supplementary materials

Supplementary material 1 Taxa and NCBI GenBank accessions used to estimate phylogeny of Spermacoceae for this study Data type Excel datasheet https://binary.pensoft.net/file/820338This dataset is made available under the Open Database License (http://opendatacommons.org/licenses/odbl/1.0/). The Open Database License (ODbL) is a license agreement intended to allow users to freely share, modify, and use this Dataset while maintaining this same freedom for others, provided that the original source and author(s) are credited.Bui Hong Quang, Khang Sinh Nguyen, Tuan Anh Le, Le Thi Mai Linh, Quoc Luan Nguyen, Duy Hoang Vu Ngo, Lei Wu, Suman Neupane

Supplementary material 2 A concatenated nexus alignment of four DNA regions (ITS, ETS, *petD*, *rps16*) used to estimate phylogeny of Spermacoceae for this study Data type Nexus alignment https://binary.pensoft.net/file/820339This dataset is made available under the Open Database License (http://opendatacommons.org/licenses/odbl/1.0/). The Open Database License (ODbL) is a license agreement intended to allow users to freely share, modify, and use this Dataset while maintaining this same freedom for others, provided that the original source and author(s) are credited.Bui Hong Quang, Khang Sinh Nguyen, Tuan Anh Le, Le Thi Mai Linh, Quoc Luan Nguyen, Duy Hoang Vu Ngo, Lei Wu, Suman Neupane

Supplementary material 3 Output of RAxML tree analysis with bootstrap values (1000 replicates) Data type Newick tree file https://binary.pensoft.net/file/820340This dataset is made available under the Open Database License (http://opendatacommons.org/licenses/odbl/1.0/). The Open Database License (ODbL) is a license agreement intended to allow users to freely share, modify, and use this Dataset while maintaining this same freedom for others, provided that the original source and author(s) are credited.Bui Hong Quang, Khang Sinh Nguyen, Tuan Anh Le, Le Thi Mai Linh, Quoc Luan Nguyen, Duy Hoang Vu Ngo, Lei Wu, Suman Neupane
